# The effects and mechanisms of low-carbon transition promoting air quality improvement: based on empirical evidence of low-carbon city pilot policy

**DOI:** 10.3389/fpubh.2026.1799779

**Published:** 2026-06-11

**Authors:** Chenxi Deng, Bingnan Guo

**Affiliations:** School of Humanities and Social Sciences, Jiangsu University of Science and Technology, Zhenjiang, China

**Keywords:** air quality, green economic efficiency, industrial structure upgrading, infrastructure level, low-carbon city pilot policy

## Abstract

In the context of the “Dual Carbon” strategic goals, promoting a comprehensive green transition of the economy and society has become a national strategic priority. Using the low-carbon city pilot policy as a quasi-natural experiment, this study empirically analyzes its impact on urban air quality and the underlying mechanisms. Based on panel data from 282 prefecture-level cities in China from 2008 to 2022, a multi-period difference-in-differences (DID) model is constructed. The results indicate that the low-carbon city pilot policy significantly improves urban air quality, a finding that remains robust after a series of rigorous tests. The policy primarily achieves this improvement by enhancing green economic efficiency and promoting industrial structure upgrading. Furthermore, the positive effects are more pronounced in non-provincial capital cities, larger cities, cities with greater local government fiscal investment and cities in high-energy-consumption regions. Interestingly, the level of urban infrastructure plays a negative moderating role; the more developed the infrastructure, the weaker the marginal improvement effect of the policy. Based on these findings, this study proposes policy recommendations, including implementing differentiated strategies and encouraging enterprises to actively pursue green transformation to achieve sustained air quality improvement.

## 1 Introduction

Air pollution represents a critical environmental challenge that constrains sustainable urban development and compromises residents’ health and well-being in China (1, 2). The effectiveness of its governance is directly linked to the nation’s ecological civilization construction and the achievement of high-quality development goals. Currently, China is at a pivotal stage in advancing its green and low-carbon transition. Notably, the State Council’s “14th Five-Year” Comprehensive Work Plan for Energy Conservation and Emission Reduction explicitly calls for “vigorously promoting energy conservation and emission reduction, intensifying the battle against pollution, and accelerating the establishment of an economic system for green, low-carbon, and circular development”. Enhancing atmospheric environmental quality and bolstering the resilience of urban ecosystems have thus ascended as key national strategic priorities. However, against the backdrop of ongoing industrialization and urbanization, the concentration of atmospheric pollutants in China remains high. Issues of regional and complex air pollution persist, posing severe challenges to sustained air quality improvement (3). In 2022, although the proportion of days with good air quality in cities at and above the prefecture level improved, more than 30% of these cities still exceeded the standard for average annual PM2.5 concentrations. Compound pollution remained particularly acute in the Beijing-Tianjin-Hebei region and its surrounding areas.^1^ Specifically, the carbon lock-in effect within industrial and energy structures has yet to be fundamentally resolved, and the pace of green transformation in traditional high-emission sectors remains relatively slow. Furthermore, the capacity and systems for urban environmental governance are unevenly developed across regions. Deficiencies still exist in some cities regarding pollution monitoring, regulatory enforcement, and the application of green technologies. Additionally, the increasing frequency of extreme weather events under climate change introduces new uncertainties into the dispersion and removal of urban air pollutants. These interconnected problems collectively pose significant pressure on achieving continuous air quality improvements in Chinese cities, highlighting an urgent need to explore scientific and effective policy intervention pathways.

To explore a low-carbon development model suited to national conditions, China has implemented the “National Low-Carbon City Pilot Policy” in three batches since 2010. This initiative aims to use pilot cities as testing grounds to accumulate experience for a nationwide green and low-carbon transition. Through a comprehensive set of measures—including setting carbon intensity targets, promoting low-carbon technologies, improving market-based incentive mechanisms, and strengthening capacity building—the policy guides pilot cities to undertake systematic low-carbon transformations in their industrial structure, energy systems, transportation modes, and construction sectors. Consequently, a critical question arises: as a comprehensive environmental regulation and development policy, can the Low-Carbon City Pilot effectively drive substantial improvement in urban air quality? What are the underlying mechanisms at play? A thorough investigation of these questions can provide empirical evidence for assessing the policy’s real-world environmental benefits and offer crucial insights for optimizing future policy design and promoting differentiated governance.

Academic research on the Low-Carbon City Pilot Policy primarily focuses on three areas. Regarding policy connotation and objectives, scholars generally view it as a voluntary environmental regulation tool centered on reducing energy consumption and carbon emissions, designed to help cities build low-carbon industrial systems, explore emission reduction pathways, and achieve a comprehensive socio-economic low-carbon transition (4, 5). In terms of policy effectiveness evaluation, studies confirm its positive role in promoting carbon emission reduction and enhancing carbon emission efficiency, with more pronounced effects in cities characterized by lower resource dependence, higher economic development levels, and greater political prominence (6). Furthermore, the policy’s impact exhibits significant spatial spillover effects, improving not only local carbon efficiency but also generating positive spillover effects on neighboring cities (7). At the micro-level, research has explored the policy’s influence on corporate behavior. Evidence indicates it incentivizes green innovation (8), improves firms’ total factor productivity, and enhances sustainable development performance (9). The primary mechanism is an innovation effect, whereby the policy drives green transformation by increasing corporate innovation investment and efficiency, strengthening environmental responsibility, and attracting talent agglomeration (10). Studies also find that command-and-control policy instruments play a dominant role in the pilot process (11). This aligns with the broader discussion on environmental regulation and innovation, where well-designed regulations can stimulate corporate environmental innovation and potentially boost competitiveness through an “innovation compensation” effect (12, 13), highlighting the significant influence of policy instrument design and institutional context on outcomes. As a vital indicator of human settlement environments and public health, the influencing factors and governance strategies of air quality have also garnered sustained scholarly attention. Regarding the causes and impacts of air pollution, research from an urban characteristics perspective shows that factors like urban street morphology (14), urban form (15), and suburban land surface temperature (16) significantly affect air quality. Pollution not only directly harms residents’ health and quality of life (17, 18) but also impedes economic development by exacerbating poverty in low-income areas (19) and affecting the agglomeration of innovative talent (20). Concerning air pollution control, studies suggest that new urbanization can effectively improve air quality through technological effects, structural upgrading effects, and environmental regulation effects (21). Additionally, the development and application of digital technologies can significantly enhance pollution control efficacy by enabling green innovation and optimizing production management (22, 23).

While these studies provide a solid theoretical foundation for this paper, they also leave room for further exploration. For instance, Song et al. (24) of the low-carbon city policy’s impact on air quality; however, their sample period is concentrated in the early stages of policy implementation, and their discussion of the underlying mechanisms is relatively brief. Based on this, this study employs China’s phased implementation of the Low-Carbon City Pilot Policy as a quasi-natural experiment. Based on panel data from 282 prefecture-level cities from 2008 to 2022, we construct a multi-period Difference-in-Differences model to empirically examine the policy’s impact on urban air quality. The contributions of this study are threefold. First, by leveraging panel data covering an extended period (2008–2022), this study captures both the dynamic effects and long-term impacts of the policy more comprehensively. Second, it constructs a mediation model that incorporates green economic efficiency and industrial structure upgrading to provide an in-depth analysis of the policy’s parallel pathways. By integrating these factors with the moderating variable of infrastructure level, the paper establishes a more systematic analytical framework encompassing policy effects, transmission mechanisms, and boundary conditions. Third, in the heterogeneity analysis, this study identifies the differentiated impacts of factors such as urban administrative hierarchy, city size, fiscal investment intensity, and regional energy consumption intensity. This offers a novel perspective for understanding why the effectiveness of low-carbon policies varies across cities. These contributions collectively foster a more nuanced understanding of the environmental performance of low-carbon city pilot and provide more targeted insights for subsequent policy optimization and diffusion.

## 2 Theoretical analysis and research hypotheses

The low-carbon city pilot policy represents a significant institutional innovation in China’s pursuit of green and low-carbon development. To address the increasingly severe challenges of climate change and explore a new urbanization pathway suited to China’s national conditions, the National Development and Reform Commission officially launched the low-carbon city pilot initiative in 2010. This initiative has subsequently designated pilot areas across three batches, encompassing numerous provinces and cities including Beijing, Shanghai, and Shenzhen, which gave shape to a policy implementation framework characterized by “central guidance, local leadership, and multi-stakeholder participation.” This pilot-first model aims to provide differentiated low-carbon transition pathways for cities at varying development levels.

The policy mandates pilot regions to formulate specialized development plans, establish carbon emission statistical monitoring systems, and advance low-carbon transformation through multiple measures such as industrial restructuring, energy mix optimization, and technological innovation. Since its implementation, pilot cities have actively explored various fields including industrial emission reduction, building energy efficiency, and transportation optimization, fostering distinctive low-carbon development models. Existing research indicates that this policy has achieved notable success in enhancing carbon emission efficiency, driving corporate green innovation, and optimizing industrial structure. Particularly within the current strategic context of the “dual carbon” goals, a systematic evaluation of the environmental effects of the low-carbon city pilot policy holds substantial practical significance for refining the policy framework and promoting the synergistic enhancement of pollution reduction and carbon mitigation.

### 2.1 The direct impact of the low-carbon city pilot policy on air quality improvement

As a crucial institutional arrangement at the national level for promoting green and low-carbon transition, the low-carbon city pilot policy aims to guide pilot cities in establishing an economic development model characterized by low energy consumption and low emissions through a systematic and differentiated set of policy instruments. In practice, the policy’s impact on air quality is likely realized through multiple pathways. Specifically, the policy first sets clear emission reduction targets and industrial access standards to directly restrict high-pollution activities. Pilot cities are required to meet carbon intensity reduction targets, which necessitates concrete measures in key areas such as energy consumption and industrial production. Many cities have adopted approaches such as phasing out backward production capacity, controlling total coal consumption, and promoting clean energy substitution, thereby reducing the generation of atmospheric pollutants (25) and producing tangible improvements in air pollution. Simultaneously, through measures such as establishing special support funds, offering tax incentives, and implementing pilot carbon emission trading schemes, the policy alters the relative returns on green investments versus traditional investments (26). This shift directs more social capital toward energy conservation, environmental protection, clean energy, and other related fields, promoting the optimization and upgrading of urban energy and industrial structures. More importantly, the long-term low-carbon infrastructure resulting from industrial restructuring also creates conditions for sustained air quality improvement.

Furthermore, the implementation of the policy gradually reshapes the behavioral patterns of both enterprises and the public. By establishing carbon emission monitoring platforms, promoting the development of low-carbon communities, and strengthening public education and outreach, the concept of low-carbon development is increasingly integrated into all levels of society. This subtle yet persistent influence helps shift societal awareness and behavioral choices, fostering a collaborative governance environment with multi-stakeholder participation (27). Air quality improvement thus becomes not merely a governmental responsibility, but a common goal pursued by the whole society. Based on the above analysis, the low-carbon city pilot policy essentially establishes a comprehensive governance framework encompassing administrative constraints, economic incentives, and social guidance. This framework operates across different dimensions and collectively contributes to positive effects on urban air quality. Therefore, this paper proposes:

*H1*: The low-carbon city pilot policy can improve urban air quality.

### 2.2 The indirect impact of the low-carbon city pilot policy on air quality improvement

#### 2.2.1 Enhancing green economic efficiency

Green economic efficiency serves as a key indicator for assessing the coordination between economic development and environmental protection. Its core connotation lies in evaluating a city’s capacity to generate economic value under given resource and environmental constraints, emphasizing the maximization of economic output with minimal environmental cost. Enhancing green economic efficiency implies that enterprises can create greater economic value with less resource consumption and pollution emission. Specifically, improvements in green economic efficiency are often accompanied by an optimized energy consumption structure, including an increased share of clean energy. This fundamentally reduces pollutant emissions from fossil fuel combustion, lowers the pollution intensity of activities such as industrial production and energy consumption from the source, and thus plays a direct role in improving air quality. Furthermore, as an institutional arrangement designed to drive green transition, the low-carbon city pilot policy provides robust support for enhancing green economic efficiency (28). The policy is not a simple administrative decree but rather a composite mechanism combining “forcing” and “incentivizing” elements. By setting clear carbon emission reduction targets, it compels enterprises to re-evaluate their production functions and internalize environmental costs (29, 30). Concurrently, market-based instruments such as fiscal subsidies and green finance provide positive incentives for enterprises to adopt energy-saving and environmentally friendly technologies. Under this dual pressure, the optimal strategy for enterprises shifts from end-of-pipe treatment to the green transformation of the entire production process. This, in turn, facilitates the reallocation of resources toward sectors with lower energy consumption and higher output. The ultimate result is a comprehensive improvement in green economic efficiency, enabling cities to support higher-quality economic output with reduced energy consumption and pollution emissions. This, at its source, undermines the formation basis of PM2.5 and contributes to improved urban air quality (31). Based on this reasoning, this paper proposes:

*H2*: The low-carbon city pilot policy improves urban air quality by enhancing urban green economic efficiency.

#### 2.2.2 Promoting industrial structure advancement

Industrial structure upgrading refers to the process through which an economic structure evolves from reliance on traditional industries dependent on resource inputs and high pollution emissions toward a modern industrial system characterized by technological innovation, knowledge intensity, and environmental friendliness. Its core feature lies in the systematic improvement of the overall industrial sector in terms of technological sophistication, value-added, and green and low-carbon performance (32). Industrial structure upgrading exerts a significant influence on air quality improvement. When traditional industries adopt green technological transformations in response to environmental constraints, they can directly reduce pollution emission intensity at the production stage (33). As low-pollution service sectors and high-tech industries come to dominate the economy, the dependency of economic growth on resources and the environment decreases, thereby facilitating a decoupling between economic growth and pollution emissions at the macro level. Furthermore, industrial structure optimization reshapes industrial linkages, encouraging upstream and downstream enterprises to form synergistic emission reduction efforts around common green standards. This kind of supply chain-based collaborative governance yields environmental benefits that far exceed isolated improvements by individual firms. However, the transformation and upgrading of industrial structure face challenges such as high transition costs, inadequate institutional safeguards, and insufficient supply of key factors including relevant technologies, talent, and capital. These constraints make it difficult for industrial transformation to be fully realized. The low-carbon city pilot policy offers an effective solution to this dilemma. The industrial structure upgrading driven by the pilot policy is not merely about increasing the share of the service sector; rather, it represents a profound qualitative transformation of the economic system from a high-carbon, extensive model toward a green, intensive one. This process is primarily achieved through a dual pathway. On one hand, by establishing stringent environmental access thresholds and carbon emission constraints, the policy exerts a strong crowding-out effect on traditional high-energy-consumption industries, compelling them either to upgrade through green technological transformation or to exit the market in an orderly manner (34). On the other hand, the clear expectations for low-carbon transition create development space for emerging green industries such as new energy, energy conservation, and environmental protection, guiding the accelerated agglomeration of key factors including capital, technology, and talent toward these sectors. This structural reshaping—which pursues both the transformation of existing industries and the cultivation of new ones—not only optimizes the proportional relationships among industries but also promotes a comprehensive green transformation within industries, spanning from production methods to value chains. As the level of industrial structure upgrading improves, the dependence of urban economic growth on highly polluting, energy-intensive industries gradually declines, and the energy consumption structure shifts toward cleaner sources. This fundamentally reduces the generation and emission of atmospheric pollutants, ultimately leading to sustained improvements in air quality. Based on this analysis, this paper proposes:

*H3*: The low-carbon city pilot policy improves urban air quality by promoting industrial structure upgrading.

#### 2.2.3 The moderating role of infrastructure level

Urban infrastructure level serves not only as a critical indicator of a city’s physical carrying capacity, but also as a key contextual variable in the implementation of policies. The extent of infrastructure development can influence the carbon emission structure of urban energy systems, transportation networks, and building operations, while also shaping the implementation pathways and adjustment challenges of low-carbon transition. Well-developed infrastructure can improve environmental performance through enhanced energy efficiency and optimized spatial layout (35). However, highly developed infrastructure may also lead to a “high-carbon lock-in effect,” whereby existing energy, transportation, and building systems, due to their long-term reliance on traditional high-carbon technologies, face difficulties in swiftly transitioning to low-carbon models, potentially hindering the adoption and diffusion of new technologies. The tension between retrofitting existing systems and constructing new ones may result in differentiated policy effectiveness across cities with varying levels of infrastructure.

Specifically, in cities with lower infrastructure levels, the existing systems’ dependence on high-carbon technologies is relatively weak. The implementation of low-carbon pilot policies can more easily promote the construction of new green infrastructure and the adoption of clean energy, offering greater potential for improvement and more pronounced environmental benefits. Conversely, in cities with higher infrastructure levels, despite their possibly stronger endogenous capacity for pollution control, the high costs of system transformation and the difficulty of technological retrofitting may constrain the policy’s environmental impact, often limiting it to incremental improvements within existing systems. As a result, the policy effect may diminish gradually. In other words, the level of infrastructure may attenuate the air quality improvement effect of the low-carbon pilot policy, implying that more developed infrastructure corresponds to smaller additional environmental gains from the policy. Based on the above analysis, this paper proposes:

*H4*: The level of urban infrastructure negatively moderates the effect of the low-carbon city pilot policy on air quality improvement—that is, the higher the infrastructure level, the weaker the marginal improvement effect of the policy.

## 3 Model setting and indicator description

### 3.1 Econometric modelling

As noted above, the list of low-carbon city pilot areas was announced in three batches in 2010, 2012, and 2017. Accordingly, to examine the relationship between the low-carbon city pilot policy and urban air quality, this study treats the implementation of the pilot policy as a quasi-natural experiment and constructs the following multi-period difference-in-differences (DID) model (36) based on prefecture-level city data from 2008 to 2022:

$$\mathrm{LnA}Q_{\mathrm{it}}=\beta_{0}+\beta_{1}\mathrm{DI}D_{\mathrm{it}}+\beta_{2}X_{\mathrm{it}}+\mu_{i}+\lambda_{t}+\varepsilon_{\mathrm{it}}$$ (1)

In the model, i and t represent the prefecture-level city and the year, respectively. The dependent variable $\mathrm{lnA}Q_{\mathrm{it}}$ denotes the air quality level of city i in year t . The core explanatory variable $\mathrm{DI}D_{\mathrm{it}}$ indicates whether city i was affected by the low-carbon city pilot policy in year t : it takes the value of 1 if the policy was in effect, and 0 otherwise. $X_{\mathrm{it}}$ represents a set of multidimensional control variables. $\beta_{0}$ is the constant term, while $\beta_{1}$ and $\beta_{2}$ are coefficients to be estimated. City fixed effects ( $\mu_{i}$ ) and year fixed effects ( $\lambda_{t}$ ) are included to control for time-invariant city heterogeneity and common time trends. The error term $\varepsilon_{\mathrm{it}}$ captures random disturbances.

### 3.2 Sample and data sources

This study utilizes panel data at the prefecture-level city scale. To ensure consistency across the dataset, cities designated as pilot zones that only covered specific districts or counties—rather than the entire administrative area—were excluded from the overall sample. Additionally, Bijie and Tongren were removed from the sample, as both were established as prefecture-level cities in 2011, making pre- and post-policy comparisons impractical. The final sample comprises 282 prefecture-level cities, yielding a total of 4,125 observations. The sample covers the period from 2008 to 2022. Data were sourced from the China City Statistical Yearbook, provincial and municipal statistical yearbooks, the China Energy Statistical Yearbook, and the Wind database. Considering data availability and continuity, as well as the fact that some prefecture-level statistical indicators have undergone adjustments in statistical caliber or have not yet been fully released for subsequent years (2023 and beyond), this study sets the research interval from 2008 to 2022 to ensure the completeness and consistency of the research sample. Furthermore, ending the sample period in 2022 facilitates a comprehensive examination of the medium- to long-term impacts of the low-carbon pilot policy under normalized economic conditions, while also enabling the identification and exclusion of potential interference from external shocks during special periods (e.g., 2020–2022) on the estimation results in robustness tests. To mitigate the influence of extreme values and ensure that the distribution of variables remains within a reasonable range, all continuous variables were winsorized at the 1% level.

### 3.3 Variables’ selection

#### 3.3.1 Explained variable

The explained variable in this study represents LnAQ. In environmental policy evaluation research, air quality serves as a key outcome variable for assessing the environmental benefits of policies. This study adopts air quality as the core dependent variable, aiming to empirically examine the direct impact of policies such as the low-carbon city pilot on atmospheric environmental quality.

To measure this variable accurately, this paper selects the annual average concentration of inhalable fine particulate matter (PM2.5) as the proxy indicator for air quality. A lower value of this indicator corresponds to better air quality. PM2.5 is one of the primary air pollutants, with significant adverse effects on human health and the ecological environment. Its concentration level is an internationally recognized and scientifically sound indicator of regional air pollution (37). In the specific model estimation, to ensure the robustness of the estimation results and the economic interpretability of the coefficients, this study applies a natural logarithmic transformation to the PM2.5 concentration data. This approach is motivated by two primary considerations. First, air pollutant concentrations typically follow a log-normal distribution; taking logarithms helps transform the data into an approximately normal distribution, mitigates the potential influence of extreme values on the estimation results, and satisfies the fundamental assumptions of classical linear regression models (38). Second, the coefficients in a log-linear model can be directly interpreted as elasticities or semi-elasticities, facilitating the quantification of the percentage change in air quality attributable to the low-carbon pilot policy. This practice has been widely adopted in authoritative environmental policy evaluation literature (24, 39). This transformation effectively mitigates the right-skewed distribution and heteroskedasticity issues present in the raw data, rendering the data more stationary.

In summary, this study uses the log-transformed annual average PM2.5 concentration as the quantitative measure of air quality and incorporates it into the econometric model as the dependent variable. The data for this variable are derived from high-resolution satellite remote sensing inversion, calibrated with ground monitoring station data, and aggregated to the city level using area-weighted averaging based on administrative boundaries. This ensures high objectivity and continuity of the data.

Explanatory variable. The core explanatory variable is a dummy variable indicating whether a city is designated as a low-carbon pilot city (DID). In assigning values, for cities included in the pilot program, all annual observations from the policy start year onward are coded as 1, and observations before the start year are coded as 0. To accurately define the timing of the policy shock, the following adjustments were made: First, considering the spillover effect of provincial-level pilots, all prefecture-level cities belonging to a province with low-carbon pilot status were treated as subject to the policy. Second, for cities that appeared in multiple batches of the pilot list, the year of the earliest batch in which they were included was taken as the policy start year.

Finally, since the second batch of pilot cities was announced at the end of 2012, the policy start year for these cities was set as 2013 to better capture the actual implementation and effect of the policy.

Control variables. Air quality reflects the atmosphere’s capacity to accommodate, disperse, and purify pollutants, representing the overall state of air cleanliness and health in a region. It is influenced by a combination of natural meteorological conditions, geographical environment, industrial structure, energy consumption, transportation emissions, and other factors, serving as a key indicator for assessing the health of urban living environments and the level of sustainable development. Therefore, drawing on relevant scholarly research (37), this paper selects a series of city-level influencing factors as control variables: ① Economic development level (GDP): measured by the logarithm of regional GDP per capita. ② Population density (Pop): measured by the ratio of the year-end total population to the area of the administrative region. ③ Financial development level (FD): reflecting the availability of capital for green investment and low-carbon transition, which is a key channel for the effective transmission of policies to real emission reductions. It is measured by the ratio of total deposits and loans of financial institutions to regional GDP. ④ Education attainment (Edu): measured by the ratio of the population with a college degree or above to the population aged 6 and above. ⑤ Education expenditure level (EE): measured by the ratio of education expenditure in the local general public budget to the year-end resident population. ⑥ Science and technology expenditure level (Rde): measured by the ratio of science and technology expenditure in local fiscal expenditure to the year-end resident population (Table 1).

**Table 1:** Variable definitions and descriptions.

Type	Variable name	Symbol	Description/measurement
Dependent variable	Air quality	LnAQ	Ln (PM2.5 annual average concentration)
Explanatory variable	Low-carbon city pilot policy	DID	Dummy variable (see detailed definition in the preceding section)
Control variables	Economic development level	GDP	Ln (regional GDP per capita)
	Population density	Pop	Year-end total population/administrative area (persons per km^2^)
	Financial development	FD	Total deposits & loans of financial institutions/regional GDP
	Education attainment	Edu	Number of people with a college degree or above/total population aged 6 and above
	Education expenditure level	EE	Education expenditure in the general public budget/year-end resident population
	Science & technology expenditure level	Rde	Per capita public expenditure on science and technology

#### 3.3.2 Mechanism variables

① Drawing on existing research, this study constructs a green economic efficiency (GEE) index based on the Data Envelopment Analysis (DEA) and Malmquist index methods (40). Following related literature (41), we regard GEE as a mediator through which the low-carbon city pilot policy may influence urban air quality. The rationale is that the policy can reshape enterprise behavior, enhance the level of green economic development at the firm level, and thereby contribute to improvements in urban air quality. The specific indicators used to construct the GEE are presented in Table 2. Using these indicators as inputs in a super-efficiency SBM model, the GEE can be constructed.

**Table 2:** Construction of green economic efficiency.

Category	Specific indicators	Economic meaning
Input indicators	Total fixed asset investment, Total water consumption, Total electricity consumption, Number of employed persons	Capital, resource, and labor inputs
Desirable outputs	Real GDP, Science expenditure, Education expenditure	Economic output and social development
Undesirable outputs	Industrial SO₂ emissions, Industrial soot and dust emissions, Industrial wastewater discharge	Environmental pollution costs

The indicator system constructed for green economic efficiency is scientifically well-grounded, conforming to the three-dimensional analytical framework of “inputs-desirable outputs-undesirable outputs” in environmental economics. At the input level, it encompasses the three factors of capital, resources, and labor, fully mapping the core variables of the production function. For desirable outputs, it employs real GDP to measure economic performance, while using science and education expenditures to characterize innovation development and human capital accumulation, thereby transcending the limitation of a single economic aggregate. For undesirable outputs, it incorporates gaseous, particulate, and water pollutants from industrial “three wastes,” directly quantifying negative environmental externalities and embodying the green development philosophy that “lucid waters and lush mountains are invaluable assets.” The overall logic follows the paradigm of the undesirable output SBM model. By incorporating environmental pollution as a negative output into the efficiency frontier measurement, this approach can accurately identify the resource and environmental costs incurred during the economic growth process across regions, providing a reliable empirical foundation for assessing green economic efficiency. ② Industrial structure advancement (ISA) serves as a key transmission channel linking low-carbon policies to air quality improvement, reflecting the inherent shift of an economic system from carbon-intensive and extensive growth toward green and intensive development. By raising the technological sophistication of industries and the efficiency of resource use, this transformation simultaneously promotes economic growth and reduces pollution emissions, forming an essential pathway through which policies exert environmental effects. In this study, industrial structure advancement is measured as the ratio of value-added of the tertiary industry to the city’s GDP in the same year (multiplied by 100).

### 3.4 Descriptive statistics

Table 3 presents the descriptive statistics of the main variables. Regarding the data distribution, the air quality variable (lnAQ) ranges from 2.334 to 4.661, with a mean of 3.718 and a standard deviation of 0.383. This indicates that the overall air quality across cities is relatively good, yet notable disparities exist among them. The explanatory variable, the low-carbon city pilot policy (DID), has a mean of 0.319 and a standard deviation of 0.466, ranging from 0 to 1, reflecting considerable variation in policy implementation across sample cities. As for the control variables, the average values are as follows: economic development level (GDP) 10.654, population density (Pop) 5.744, financial development level (FD) 2.446, education attainment (Edu) 0.019, education expenditure level (EE) 0.178, and science and technology expenditure level (Rde) 0.017.

**Table 3:** Descriptive statistics.

Variables	N	Mean	SD	Min	Max
LnAQ	4,125	3.718	0.383	2.334	4.661
DID	4,125	0.319	0.466	0	1
GDP	4,125	10.654	0.635	8.389	12.457
Pop	4,125	5.744	0.976	1.653	9.089
FD	4,125	2.446	1.203	0.504	21.297
Edu	4,125	0.019	0.025	0	0.147
EE	4,125	0.178	0.041	0.018	0.377
Rde	4,125	0.017	0.017	0.001	0.207

## 4 Empirical analysis

### 4.1 Baseline regression result

The benchmark regression results are presented in Table 4. Column (1) reports the results of a model that includes only the core explanatory variable and does not incorporate two-way fixed effects; the estimated coefficient is −0.2028. When only control variables are included without fixed effects, as shown in Column (3), the coefficient slightly rises to −0.1670. Column (4) presents the empirical results after introducing both control variables and two-way fixed effects, yielding a coefficient of −0.0351. The findings indicate that, regardless of whether control variables and fixed effects are included, the regression results show a statistically significant negative correlation at the 5% level. Specifically, a one-unit increase in the implementation of the low-carbon city pilot policy is associated with a 3.51% improvement in urban air quality. This robust result suggests that the low-carbon city pilot policy plays a significant role in promoting urban air quality. The underlying mechanisms can be attributed to the following aspects: first, the policy imposes binding constraints that directly limit high-emission activities; second, it employs fiscal and financial incentives to encourage green investment and technological innovation; and finally, it facilitates the transition toward low-carbon industrial and energy structures, thereby reducing pollution emissions at the source. Therefore, the observed policy effect reflects the combined outcome of direct regulation, market-based incentives, and long-term structural adjustments. Accordingly, Hypothesis 1 is supported.

**Table 4:** Baseline regression result.

Variables	(1)	(2)	(3)	(4)
	LnAQ	LnAQ	LnAQ	LnAQ
DID	−0.2028***	−0.0463***	−0.1670***	−0.0351**
	(−16.36)	(−3.23)	(−15.31)	(−2.54)
GDP			−0.1443***	−0.1227***
			(−14.53)	(−4.41)
Pop			0.2122***	−0.2031***
			(34.50)	(−4.01)
FD			−0.0920***	−0.0155**
			(−18.12)	(−2.26)
Edu			2.1412***	1.6636*
			(7.79)	(1.94)
EE			−1.3329***	−0.4723***
			(−9.82)	(−2.81)
Rde			−2.2125***	−1.3783***
			(−5.82)	(−3.93)
Constant	3.7826***	3.7327***	4.5484***	6.3154***
	(540.58)	(815.66)	(40.70)	(14.40)
N	4,125	4,125	4,125	4,125
R^2^	0.0610	0.8967	0.3392	0.9023
Year FE	No	Yes	No	Yes
Id FE	No	Yes	No	Yes

***, **, and * denote significance at the 1, 5, and 10% levels, respectively; t-statistics are in parentheses.

### 4.2 Robustness tests

#### 4.2.1 Parallel trend test

**Figure 1:**
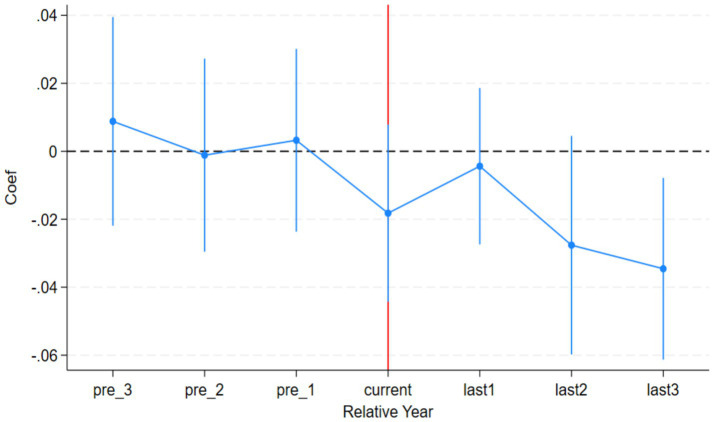
Parallel trend test.

The Difference-in-Differences (DID) model requires the parallel trends assumption to hold. This study employs a panel event-study approach to test this assumption. The year of policy implementation (current) is set as the baseline period, and its coefficient is normalized to 0 to isolate the dynamic effects of the policy. Therefore, the policy effect should be identified by comparing the post-policy coefficients (last1–last3) with the pre-policy trend (pre_1), rather than by directly interpreting the current coefficient. Figure 1 plots the estimated coefficients along with their 95% confidence intervals for three years before and after the implementation of the low-carbon city pilot policy, using the implementation year as the baseline. The results show that from three years before the policy (pre_3) to one year before (pre_1), the trends in the PM2.5 index between the treatment and control groups remained largely parallel, with coefficients close to zero and statistically insignificant, satisfying the parallel trends assumption. After the policy implementation, the effect coefficients turn negative but are not statistically significant in the implementation year (current) or the following two years (last1, last2). It is not until the third year after implementation (last3) that the negative effect becomes statistically significant. This indicates that the low-carbon pilot policy’s improvement effect on air quality exhibits a lag of approximately 2–3 years, after which a significant and sustained promoting effect emerges. Therefore, the low-carbon city policy exerts a persistent and gradually strengthening long-term effect on urban air quality.

#### 4.2.2 Placebo test

Although the baseline regression results verify the positive impact of the low-carbon city pilot policy on urban air quality, the estimates may still be subject to bias due to omitted variables or other potential confounding factors. To exclude potential bias in the baseline regression results caused by possible omission of certain important variables or other potential disturbances, this study conducts a counterfactual estimation following the approach of related research (42).

**Figure 2:**
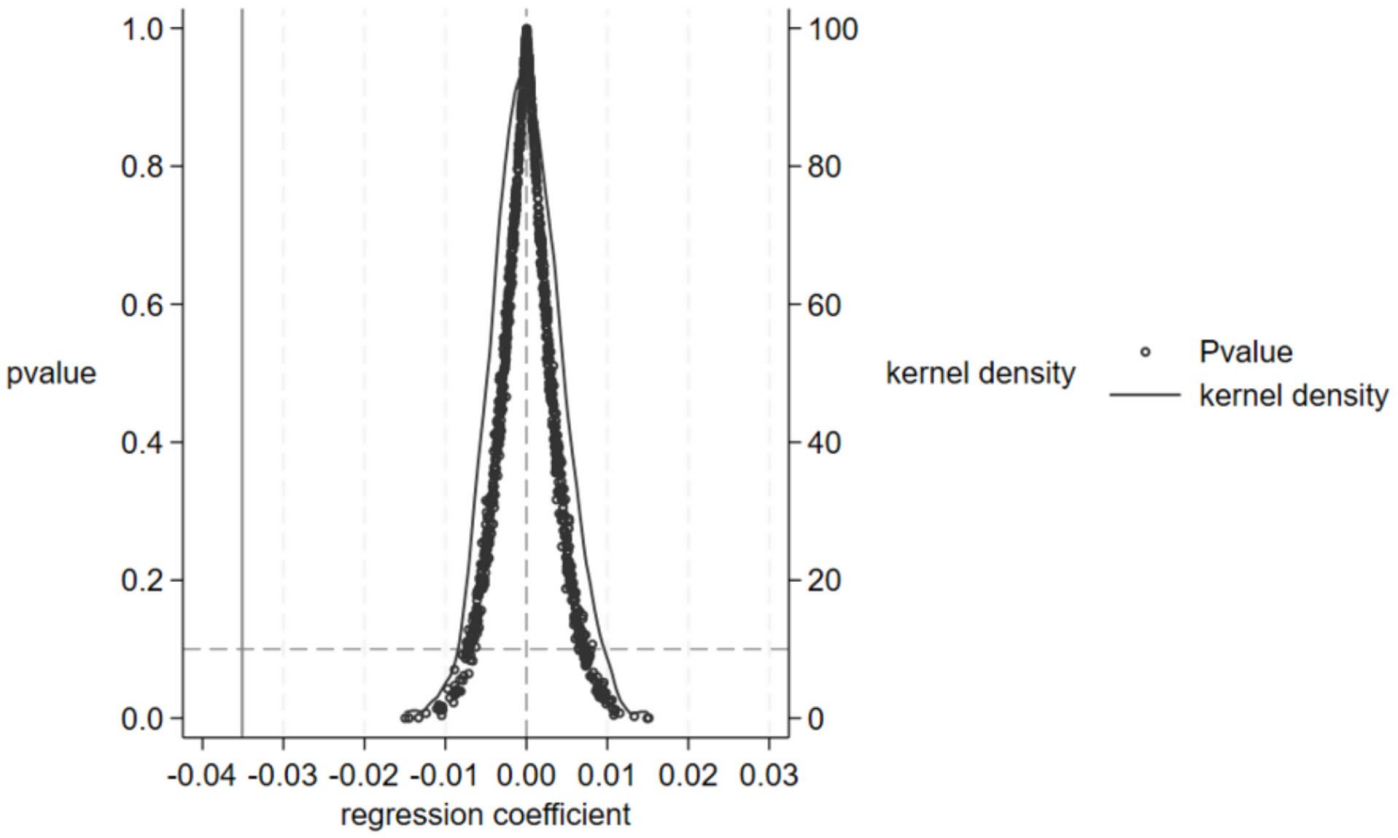
Placebo test.

This study adopts a random sampling method to randomly shuffle the establishment of the low-carbon city pilot policies and reassign them to a certain city, thereby constructing a placebo treatment variable to replace the DID in Equation 1. The regression is re-estimated, and this procedure is repeated 1,000 times to enhance the credibility of the results. Theoretically, the estimated coefficient of the placebo treatment variable should not be significantly different from zero. The curve in Figure 2 represents the kernel density distribution and the estimated coefficient function from the 1,000 repetitions, while the hollow circles denote the *p*-values corresponding to the estimated coefficients. The true estimated coefficient from the baseline regression in the main text (−0.0351) deviates significantly from the placebo coefficients generated by the random test. It can be seen that the placebo regression coefficients in Figure 2 are generally distributed around zero with a normal distribution, whereas the true coefficient of DID in the baseline regression lies clearly outside the kernel density function, further confirming the credibility of the findings.

#### 4.2.3 Entropy balancing-DID

Since the selection of low-carbon pilot cities was not entirely random, sample selection bias may exist. Conventional propensity score matching (PSM) requires the common support assumption to hold; however, in this study, systematic differences in baseline characteristics—such as population density and financial development level—between the treatment and control groups lead to unsatisfactory matching performance. To address this, entropy balancing is employed to reweight the control group so that the mean values of the baseline covariates exactly match those of the treatment group, thereby effectively mitigating selection bias. Column (1) of Table 5 reports the DID regression results after entropy balancing. The balancing was performed using pre-policy cross-sectional data from 2009, with covariates including economic development level, population density, financial development level, education attainment, education expenditure level, and science and technology expenditure level. After reweighting, the coefficient of the core explanatory variable DID is −0.0301 and significant at the 10% level, indicating that the low-carbon city pilot policy reduces PM2.5 concentration by approximately 3%. This estimate is highly consistent with the baseline regression result, demonstrating that the policy effect remains robust after controlling for sample selection bias. The model R^2^ is 0.8991, suggesting that the control variables collectively possess strong explanatory power for PM2.5. Table 6 compares the covariates before and after entropy balancing. Prior to balancing, significant differences existed between the treatment and control groups in variables such as population density and financial development level. After entropy balancing, the standardized differences for all covariates drop to 0.0%, satisfying the equal-moment conditions and further confirming the reliability of the results.

**Table 5:** Robustness test results.

Variables	(1)	(2)	(3)	(4)
	Entropy balancing-DID	Excluding the pandemic period	Excluding municipalities	Excluding interference from other policies
DID	−0.0301*	−0.0272*	−0.0324**	−0.0482***
	(−1.69)	(−1.83)	(−2.37)	(−3.04)
GDP	−0.1022***	−0.1481***	−0.1223***	−0.1023***
	(−3.37)	(−4.23)	(−4.54)	(−3.09)
Pop	−0.2094***	−0.2546***	−0.1988***	−0.2374***
	(−3.53)	(−3.27)	(−4.04)	(−2.93)
FD	−0.0135	−0.0117**	−0.0158**	−0.0094
	(−1.21)	(−2.09)	(−2.34)	(−0.91)
Edu	2.5101*	1.5808	1.6102*	1.5695
	(1.90)	(1.64)	(1.93)	(1.35)
EE	−0.4272**	−0.4830***	−0.4580***	−0.2671
	(−2.28)	(−2.74)	(−2.79)	(−1.28)
Rde	−1.1898***	−1.1099***	−1.3814***	−1.2687***
	(−2.85)	(−2.95)	(−4.02)	(−2.90)
Constant	6.8468***	6.9582***	6.3017***	6.2273***
	(12.81)	(13.20)	(14.92)	(10.13)
N	4,011	3,320	4,081	2,681
R^2^	0.8991	0.0623	0.0625	0.0615
Year FE	Yes	Yes	Yes	Yes
Id FE	Yes	Yes	Yes	Yes

***, **, and * denote significance at the 1, 5, and 10% levels, respectively; t-statistics are in parentheses. R^2^ refers to within R-squared.

**Table 6:** Effectiveness test of entropy balancing.

Matching Stage	Variables	Treat			Control			Standardized bias (%)
		Mean	Var	Skewness	Mean	Var	Skewness	
Before	GDP	10.2100	0.3912	−0.0413	9.9760	0.3357	0.5599	38.8%
	Pop	5.8920	0.8482	−0.0426	5.6410	0.9353	−1.107	26.6%
	FD	2.3680	1.3570	1.5880	1.7620	0.5222	2.6090	62.5%
	Edu	0.0223	0.0007	1.8200	0.0116	0.0002	3.3670	48.4%
	EE	0.1791	0.0022	0.2003	0.1882	0.0018	0.0719	−20.2%
	Rde	0.0168	0.0002	1.944	0.0110	0.0001	2.6380	48.4%
After	GDP	10.2100	0.3912	−0.0413	10.2100	0.3650	−0.0878	0%
	Pop	5.8920	0.8482	−0.0426	5.8920	0.7615	−1.345	0%
	FD	2.3680	1.3570	1.5880	2.3680	2.0120	1.879	0%
	Edu	0.0223	0.0007	1.8200	0.0223	0.0007	1.68	0%
	EE	0.1791	0.0022	0.2003	0.1791	0.0018	0.0015	0%
	Rde	0.0168	0.0002	1.9440	0.0168	0.0002	1.887	0%

Compared with PSM, entropy balancing does not require the common support assumption and ensures exact equality of specified moment conditions between the treatment and control groups, thereby avoiding sample loss due to matching failure. This method is particularly suitable in contexts where structural differences exist between the groups, enhancing the credibility of the estimated results.

#### 4.2.4 Excluding specific time periods

Given that the COVID-19 pandemic constitutes an unpredictable exogenous shock, to eliminate potential structural disruptions it may have caused, this study restricts the sample period to the pre-pandemic years 2008–2019 and re-estimates the regression. The results are reported in Column (2) of Table 5. The core explanatory variable remains statistically significant with an unchanged sign, indicating that the baseline findings are not driven by this exceptional event. This further confirms the robustness of the benchmark regression results.

#### 4.2.5 Excluding specific spatial samples

To avoid potential estimation bias arising from the disproportionately large economic and financial scale of direct-controlled municipalities, the samples of Beijing, Shanghai, Tianjin, and Chongqing are removed from the dataset, and the model is re-estimated. The results are presented in Column (3) of Table 5. The sign and significance of the coefficient on the low-carbon city pilot policy remain unchanged, providing strong evidence that the baseline conclusion is not influenced by specific sample attributes and possesses good robustness.

#### 4.2.6 Excluding interference from other policies

In addition to the low-carbon city pilot policy, the smart city pilot policy can also reduce air pollution levels. To isolate the potential confounding interference of the smart city pilot policy on air pollution and avoid confusing its effects with those of the low-carbon city pilot policy, this study excludes all smart city pilot cities (regardless of whether they are also low-carbon pilot cities) and retains only the sample of non-smart city pilot cities, then re-estimates the baseline model. The results are shown in Column (4) of Table 5. The sign and significance of the coefficient of the core explanatory variable—the low-carbon city pilot policy—remain substantially unchanged, indicating that the baseline findings are not driven by the smart city pilot policy and possess good robustness.

### 4.3 Heterogeneity analysis

#### 4.3.1 Provincial capitals vs. non-capital cities

While the baseline regression reveals the average treatment effect, heterogeneity across cities may be masked. Given the systematic differences among Chinese cities in administrative rank, resource endowment, and development stage, and following related studies (43), this paper further examines whether the policy effect varies between provincial capitals and non-capital cities. The sample is therefore split accordingly for subgroup analysis. The results are reported in Columns (1) and (2) of Table 7. The coefficient for provincial capitals is statistically insignificant, whereas the coefficient for non-capital cities is significantly negative at the 10% level, indicating that the policy leads to a significant improvement in air quality in non-capital cities. The empirical *p*-value for the difference in regression coefficients between groups, obtained via the Bootstrap method with 1,000 repetitions, is 0.008, confirming that the DID regression coefficients are significantly different between provincial capital cities and non-capital cities. The likely explanation is that provincial capitals have typically been focal points of environmental regulation even before the pilot policy, with measures such as industrial relocation and energy-structure optimization initiated earlier. Consequently, their baseline air quality is relatively better, leaving limited marginal room for further improvement from the additional pilot policy. The reason why non-provincial capital cities exhibit a more pronounced improvement in air quality under the low-carbon pilot policy lies fundamentally in the substantial “regulatory gap” formed between their initially lower levels of environmental governance and the stringent assessments imposed after policy intervention. Prior to the pilot, these cities often compromised environmental enforcement to some extent in pursuit of economic growth, and even undertook polluting industries relocated from provincial capitals. This resulted not only in poorer baseline air quality but also in a relatively heavy industrial structure with significant historical environmental debt. The introduction of the low-carbon pilot policy disrupted this pattern. By incorporating hard environmental targets into local government performance assessments, the policy compelled these cities to rapidly tighten enforcement efforts and undertake targeted adjustments to address their overly heavy industrial structures. This “catch-up effect,” driven by policy pressure, granted non-capital cities-originally situated in environmental regulatory lowlands-greater leeway for marginal improvement. Consequently, they demonstrate statistically more significant policy effects compared to provincial capitals.

**Table 7:** Heterogeneity test results.

Variables	Provincial capitals		City-size heterogeneity		Fiscal investment heterogeneity		Energy consumption intensity heterogeneity	
	Provincial capitals	Non-capital cities	Large cities	Small-medium cities	High intensity	Low intensity	High-energy-consumption regions	Low-energy-consumption regions
	(1)	(2)	(3)	(4)	(5)	(6)	(7)	(8)
DID	−0.0287	−0.0286*	−0.0337**	−0.0209	−0.0282*	−0.0305	−0.0745***	−0.0210
	(−0.83)	(−1.91)	(−2.25)	(−0.87)	(−1.75)	(−1.28)	(−2.89)	(−1.27)
GDP	−0.2557**	−0.1168***	−0.0765**	−0.1402***	−0.1365***	−0.1278***	−0.1455***	−0.0851
	(−2.31)	(−4.21)	(−2.20)	(−3.58)	(−3.34)	(−3.82)	(−3.63)	(−1.57)
Pop	−0.0054	−0.2801***	−0.1553*	−0.2185***	−0.0984	−0.3088***	−0.1841***	−0.0236
	(−0.05)	(−4.87)	(−1.92)	(−2.96)	(−1.43)	(−3.82)	(−2.92)	(−0.33)
FD	−0.0307	−0.0143**	−0.0283***	−0.0099	−0.0350***	−0.0071	−0.0165*	−0.0247*
	(−1.47)	(−2.06)	(−2.84)	(−1.46)	(−2.75)	(−1.26)	(−1.83)	(−1.92)
Edu	2.0590	0.4388	1.7830	1.4653	1.6267	1.0130	1.6964	1.1136
	(1.47)	(0.37)	(1.61)	(1.20)	(1.43)	(0.69)	(1.51)	(0.81)
EE	−1.2583	−0.3822**	−0.6344***	−0.3766	−0.6275***	−0.2891	−0.5170**	−0.4069**
	(−1.89)	(−2.25)	(−3.11)	(−1.56)	(−2.64)	(−1.50)	(−2.11)	(−2.10)
Rde	−1.0200	−1.4128***	−0.9923***	−1.3434**	−0.5957*	−2.2249***	−1.9733***	−1.0270***
	(−1.47)	(−3.58)	(−2.65)	(−2.47)	(−1.76)	(−4.49)	(−3.10)	(−2.97)
Constant	6.9337***	6.6932***	5.8260***	6.3759***	6.0688***	6.8130***	6.4285***	4.9575*
	(4.86)	(14.28)	(8.10)	(11.81)	(10.03)	(11.23)	(11.08)	(6.34)
N	430	3,581	1980	2027	2040	1940	2000	2,103
R^2^	0.1543	0.0660	0.0569	0.0584	0.0404	0.0985	0.0797	0.0215
Id FE	Yes	Yes	Yes	Yes	Yes	Yes	Yes	Yes
Year FE	Yes	Yes	Yes	Yes	Yes	Yes	Yes	Yes

***, **, and * denote significance at the 1, 5, and 10% levels, respectively; t-statistics are in parentheses.

#### 4.3.2 City-size heterogeneity

Considering the notable differences in resource endowment, governance capacity, and emission structure across cities of different sizes, this study tests whether the effect of the low-carbon pilot policy varies with city tier. Based on the median of permanent resident population, the sample is divided into “large cities” and “small-medium cities” for separate regressions. The Chow test yields a *p*-value of 0.000 and is statistically significant at the 1% level, providing strong evidence that the impact mechanisms differ substantially across city-size groups. The results are presented in Columns (3) and (4) of Table 7. In the large-city group, the coefficient of the policy variable DID is significantly negative at the 5% level, indicating a clear improvement in air quality attributable to the policy. For small-medium cities, the coefficient is negative but statistically insignificant. This divergence aligns with practical logic and reflects a scale-dependent policy effect. The more pronounced policy effect in large cities stems from their efficient resource allocation capacity and the economies of scale in pollution control. Large cities are often confronted with more stringent environmental capacity constraints and higher public environmental demands, which endow local governments with stronger political will and fiscal capacity to drive structural transformation when implementing low-carbon pilots. Compared to small and medium-sized cities, large cities can leverage their agglomeration advantages to deploy advanced pollution monitoring networks, promote clean energy technologies, and phase out energy-intensive industries at lower unit costs. Furthermore, the industrial system in large cities is relatively complete, and the effects of technological innovation and spillover are more evident. This enables low-carbon policies to rapidly permeate all aspects of production and daily life, forming a systematic synergy for emission reduction and yielding immediate and substantial aggregate policy dividends in terms of air quality improvement.

These findings imply that the current effectiveness of the low-carbon pilot policy is primarily concentrated in large cities with mature governance systems and strong economic capacity. To extend low-carbon development to smaller cities in the future, it will be necessary to enhance their environmental governance capabilities and provide differentiated fiscal and technical support, ensuring the policy is adapted to local conditions.

#### 4.3.3 Heterogeneity in fiscal investment intensity

To further explore the heterogeneous impact of the low-carbon city pilot policy, this study uses the logarithm of total government expenditure on energy conservation and environmental protection (after adding 1 to mitigate right-skewness) as a measure of fiscal investment intensity. The sample is split at the median into high-intensity and low-intensity groups for regression analysis. Similarly, the Chow test was employed to examine the heterogeneity across groups with different fiscal investment intensity, and the result was significant at the 1% level, which formally establishes significant heterogeneity in the DID coefficients between cities with high and low fiscal investment intensity. Columns (5) and (6) of Table 7 report the results. In cities with higher fiscal investment intensity, the policy shows a significantly negative effect on PM2.5 at the 10% level. In cities with lower intensity, the coefficient is negative but statistically insignificant. This suggests that the effectiveness of the policy depends critically on the local government’s fiscal implementation capacity. The difference highlights a “resource constraint” in policy transmission. Cities with greater fiscal investment can provide essential supporting funding and infrastructure for low-carbon transition, thereby translating macro-policy goals into concrete micro-level actions. Conversely, cities with weaker fiscal resources may be unable to support comprehensive and in-depth implementation, leaving the policy effect largely nominal. This finding offers a clear policy implication: when promoting low-carbon pilots, the central government should establish incentive-compatible fiscal support mechanisms. For pilot cities with limited fiscal capacity, stronger vertical fiscal transfers or dedicated green financing instruments should be provided to alleviate resource constraints, ensure the universal achievement of policy goals, and avoid a new gap in policy effectiveness due to fiscal disparity.

#### 4.3.4 Heterogeneity in regional energy consumption intensity

Considering that differences in industrial structure across cities may lead to heterogeneous effects of the low-carbon pilot policy between high-energy-consumption and low-energy-consumption regions, potentially affecting the reliability of the research findings, this study divides the sample cities into high and low energy consumption regions for heterogeneity testing. Total energy consumption is calculated based on three components—total electricity consumption, coal gas and natural gas supply, and liquefied petroleum gas supply—using standard coal equivalent conversion coefficients. Cities are then classified according to the median of energy consumption intensity. The bootstrap test with 1,000 replications yields a *p*-value of less than 0.001, indicating that the DID treatment effects vary significantly across cities with different levels of energy consumption intensity. Columns (7) and (8) of Table 7 report the heterogeneity regression results for different levels of energy consumption intensity. The regression results reveal that the low-carbon city pilot policy has a significant improving effect on air quality in high-energy-consumption regions at the 1% level, while its effect in low-energy-consumption regions is statistically insignificant. This finding may be explained by the following mechanisms. High-energy-consumption regions typically bear heavier industrial functions, with energy consumption structures dominated by fossil fuels such as coal, resulting in higher pollutant emission intensity per unit of output. Consequently, these regions face greater emission reduction pressure and have more room for adjustment. The implementation of the low-carbon pilot policy—by forcing industrial structure upgrading and promoting clean energy substitution—can rapidly release substantial emission reduction dividends, thereby bringing about significant improvements in air quality. In contrast, low-energy-consumption regions have often already undergone industrial transformation or energy structure optimization, with relatively low baseline pollution levels. The marginal room for improvement brought by policy intervention is therefore limited, rendering the policy effect statistically insignificant.

### 4.4 Mechanism test

Following the theoretical hypotheses and analytical framework established earlier, the low-carbon city pilot policy can improve urban air quality by enhancing urban green economic efficiency and advancing the industrial structure. To verify these mechanism pathways, a mediation effect model based on the stepwise regression approach proposed by Baron and Kenny (44) is constructed, as shown in Equations (2), (3) and (4):

$$\mathrm{LnA}Q_{\mathrm{it}}=\beta_{0}+\beta_{1}\mathrm{DI}D_{\mathrm{it}}+\beta_{2}X_{\mathrm{it}}+\mu_{i}+\lambda_{t}+\varepsilon_{\mathrm{it}}$$ (2)

$$\begin{array}{c} \mathrm{Me}d_{\mathrm{it}}=\alpha_{0}+\alpha_{1}\mathrm{DI}D_{\mathrm{it}}+\alpha_{2}X_{\mathrm{it}}+\mu_{i}+\lambda_{t}+\sigma_{\mathrm{it}} \end{array}$$ (3)

$$\begin{array}{c} \mathrm{LnA}Q_{\mathrm{it}}=\rho_{0}+\rho_{1}\mathrm{DI}D_{\mathrm{it}}+\rho_{2}\mathrm{Me}d_{\mathrm{it}}+\rho_{3}X_{\mathrm{it}}+\mu_{i}+\lambda_{t}+\varsigma_{\mathrm{it}} \end{array}$$ (4)

Here, the mediator variables ( Med ) are green economic efficiency ( GEE ) and industrial structure advancement ( ISA ). All other variables remain consistent with the baseline model specification. Table 8 presents the results of the mediation effect test using the stepwise regression method. Columns (1) to (3) report the test for the mediation mechanism via green economic efficiency. The results reveal that the low-carbon city pilot policy not only significantly reduces urban PM2.5 concentration (thereby improving air quality) with a total effect of −0.0302, but also effectively enhances urban green economic efficiency. A deeper analysis indicates a positive correlation between the policy and green economic efficiency. The Sobel test confirms that the policy indirectly lowers PM2.5 concentration and improves air quality by elevating green economic efficiency. This can be attributed to the fact that the low-carbon city pilot policy, through measures such as optimizing the industrial structure, guiding enterprises to adopt cleaner production technologies, and strengthening resource recycling, enhances the green development level and resource-environment coordination of the urban economy—i.e., it improves green economic efficiency. This improvement implies a reduction in the resource and environmental cost per unit of economic growth, thereby cutting pollution emissions at the source and notably suppressing air pollutants like PM2.5. Consequently, green economic efficiency serves not only as a key intermediary channel for policy transmission but also elucidates the synergistic pathway between low-carbon city development, high-quality economic growth, and ecological improvement. These findings robustly support Hypothesis 2.

**Table 8:** Mediation effect test.

Variables	(1)	(2)	(3)	(4)	(5)	(6)
	LnAQ	GEE	LnAQ	LnAQ	ISA	LnAQ
DID	−0.0302**	0.0145*	−0.0268**	−0.0302**	−0.9594**	−0.0321**
	(−2.21)	(1.81)	(−1.97)	(−2.21)	(−2.11)	(−2.37)
GEE			−0.2504***			
			(−3.80)			
ISA						−0.0020*
						(−1.84)
GDP	−0.1217***	−0.0452***	−0.1314***	−0.1217***	−4.0108***	−0.1292***
	(−4.55)	(−3.53)	(−4.84)	(−4.55)	(−4.17)	(−4.80)
Pop	−0.2301***	0.0774***	−0.2120***	−0.2301***	−3.9564**	−0.2383***
	(−4.75)	(3.06)	(−4.47)	(−4.75)	(−2.53)	(−4.83)
FD	−0.0145**	−0.0064*	−0.0162**	−0.0145**	0.7738***	−0.0131**
	(−2.23)	(−1.91)	(−2.36)	(−2.23)	(2.64)	(−2.07)
Edu	1.7317**	−0.1471	1.6951**	1.7317**	22.3703	1.7793**
	(2.07)	(−0.35)	(2.06)	(2.07)	(1.12)	(2.13)
EE	−0.4384***	0.1011	−0.3995**	−0.4384***	18.6887***	−0.4035**
	(−2.68)	(1.15)	(−2.38)	(−2.68)	(3.65)	(−2.55)
Rde	−1.3386***	0.0035	−1.3311 ***	−1.3386***	20.2475	−1.2991***
	(−3.94)	(0.02)	(−4.01)	(−3.94)	(1.60)	(−3.80)
Constant	6.4839***	0.4408**	6.5833***	6.4839***	101.4212***	6.6852***
	(15.44)	(2.16)	(15.80)	(15.44)	(7.51)	(15.34)
N	4,011	3,986	3,986	4,011	4,007	4,007
R^2^	0.0658	0.0514	0.0736	0.0658	0.0802	0.0689
Year FE	Yes	Yes	Yes	Yes	Yes	Yes
Id FE	Yes	Yes	Yes	Yes	Yes	Yes

***, **, and * denote significance at the 1, 5, and 10% levels, respectively; t-statistics are in parentheses.

Similarly, Columns (4) to (6) of Table 8 demonstrate how the low-carbon city pilot policy affects urban air quality through industrial structure advancement. The results show that the policy significantly reduces the indicator for industrial structure advancement (measured as the share of tertiary industry) at the 5% level. Contrary to the conventional expectation that low-carbon policies should raise the service sector share, this study finds the policy significantly lowers it. This does not indicate policy failure but reflects a deeper structural adjustment: the policy achieves emission reduction through cleaner industrial transformation while simultaneously crowding out high-energy-consumption service sectors, resulting in a greener, specialized structure characterized by “stronger and cleaner industry alongside lighter services.” This finding extends the Porter Hypothesis, suggesting that low-carbon policies can promote green development through qualitative upgrading rather than mere quantitative substitution.

Furthermore, industrial structure advancement significantly reduces urban PM2.5 concentration at the 10% level, thereby improving air quality. The reason is that the industrial advancement driven by low-carbon policies is qualitative rather than a simple shift in sectoral shares. Although the policy temporarily reduces the proportion of the service sector, it fosters green upgrading within industry through mandatory clean technology retrofits, while crowding out high-pollution services tied to traditional industry and spurring high-end service sectors like green technology R&D. This structural reshaping—characterized by “cleaner industry and more specialized services”—reduces the pollution intensity per unit of output from the source, leading to a significant reduction in PM2.5 emissions and improved air quality, thereby confirming Hypothesis 3.

### 4.5 Moderating effect test

The preceding analysis confirms that the low-carbon city pilot policy effectively reduces PM2.5 concentration by enhancing green economic efficiency and advancing industrial structure, revealing a general transmission pathway of the policy. However, the significant variation in policy effectiveness across cities suggests that the smoothness of this pathway may be strongly constrained by local conditions. To explore this further, this study introduces the level of urban infrastructure as a core moderating variable, as it defines the boundary conditions under which the policy operates in contexts of “incremental new construction” versus “retrofitting existing stock.” This variable captures the completeness of a city’s existing physical systems, which determines the adjustment costs and the strength of path dependence in the green transition. The infrastructure level index used here is constructed through the entropy method based on basic indicators such as per capita road area and per capita green space, and is centered in the regression analysis.

The purpose of this moderating effect test is to examine whether a city’s material foundation and carrying capacity systematically alter the green transition process triggered by the policy and its resulting environmental performance. This shifts the inquiry from “how the policy works” to “under what conditions the policy is more effective,” thereby identifying boundary conditions and providing more solid empirical evidence for differentiated and targeted low-carbon governance. The specific model is constructed as follows:

$$\begin{array}{c} \begin{array}{l} {\text{LnAQ}}_{\mathrm{it}}=\beta_{0}+\beta_{1}\mathrm{DI}D_{\mathrm{it}}+\beta_{2}IL_{\mathrm{it}}+\beta_{3}{\left(\mathrm{IL}\times \mathrm{DID}\right)}_{\mathrm{it}}\\ +\beta_{4}X_{\mathrm{it}}+\mu_{i}+\lambda_{t}+\varepsilon_{\mathrm{it}} \end{array} \end{array}$$ (5)

Table 9 reports the moderating effect of urban infrastructure level on the effectiveness of the low-carbon pilot policy. Column (1) shows that the coefficient of the interaction term DID×IL is significantly positive at the 5% level (0.0744, *p* < 0.05), indicating that the infrastructure level exerts a significant negative moderating effect on the policy outcome: the more developed a city’s infrastructure, the weaker the marginal reduction effect of the low-carbon pilot policy on PM2.5. A between-group difference test confirms that the policy effect differs significantly between cities with low and high infrastructure levels (*F* = 4.33, *p* = 0.0385). To verify this asymmetric effect, Columns (2) and (3) present subgroup regressions based on the median infrastructure level (with 17 observations lost due to missing infrastructure data). Column (2) shows that in cities with low infrastructure, the policy significantly reduces the log-concentration of PM2.5 by 0.0448, equivalent to a decrease in PM2.5 concentration of about 4.5%. In contrast, Column (3) indicates that in cities with high infrastructure, the policy effect becomes statistically insignificant and its magnitude shrinks considerably. This further confirms the asymmetry of the policy effect: the low-carbon policy produces a significant emission-reduction effect only in cities with relatively low infrastructure levels, which aligns with the law of diminishing marginal returns—cities with weaker infrastructure have greater potential for green transformation and are therefore more responsive to policy incentives.

**Table 9:** Moderating effect test.

Variables	(1)	(2)	(3)
	LnAQ	LnAQ	LnAQ
DID	−0.0325**	−0.0448**	−0.0123
	(−2.40)	(−2.56)	(−0.53)
IL	−0.0537***		
	(−2.73)		
DID×IL	0.0744**		
	(2.16)		
GDP	−0.1267***	−0.0849***	−0.0843*
	(−4.88)	(−2.65)	(−1.95)
Pop	−0.2176***	−0.3417***	−0.0989*
	(−4.51)	(−4.00)	(−1.91)
FD	−0.0101	0.0141	−0.0254*
	(−1.61)	(1.23)	(−1.79)
Edu	1.4514	2.5164**	−0.2801
	(1.65)	(2.35)	(−0.26)
EE	−0.3696**	−0.3917*	−0.3577
	(−2.17)	(−1.83)	(−1.52)
Rde	−1.3712***	−1.7939***	−0.4572
	(−4.00)	(−3.30)	(−1.27)
Constant	6.4574***	6.7166***	5.3394***
	(15.98)	(11.13)	(9.29)
N	3,913	1944	1952
R^2^	0.0661	0.1288	0.0217
Id FE	Yes	Yes	Yes
Year FE	Yes	Yes	Yes

***, **, and * denote significance at the 1, 5, and 10% levels, respectively; t-statistics are in parentheses.

Based on the regression coefficients, the threshold at which the net policy effect turns from negative to zero occurs when the centered infrastructure level (IL) reaches 0.437. Because IL is centered, this corresponds to an original infrastructure level below 1.2282. Approximately 92% of the sample cities satisfy this condition, indicating that the current significant improvement effect of the low-carbon pilot policy covers the vast majority of cities whose infrastructure level is below this specific threshold. This high coverage stems from the right-skewed distribution of infrastructure levels across Chinese cities: a small number of developed cities possess highly advanced infrastructure, while the majority remain at a moderate to low level. Consequently, the policy’s effective range encompasses most of the sample. This finding echoes the law of diminishing marginal returns: in the early stage of policy intervention, cities with weaker infrastructure—owing to larger green-transition potential and lower adjustment costs—respond more sensitively to policy incentives. In contrast, cities with well-developed infrastructure often exhibit strong technological and institutional path dependence on high-carbon patterns, leaving limited marginal room for improvement triggered by the policy.

It is worth noting that the main effect of IL is significantly negative at the 1% level (−0.0537), revealing the dual role of infrastructure. On the one hand, well-developed infrastructure directly improves air quality by enhancing energy efficiency and optimizing commuting structures. On the other hand, it simultaneously diminishes the incremental improvement potential of the low-carbon policy, resulting in diminishing marginal returns. This co-existence of a “negative main effect and a positive moderating effect” indicates that the current infrastructure system likely contains a structural tension between green and gray elements—the lock-in effect of traditional high-carbon infrastructure, such as coal-based heating networks and fuel-dependent transport, suppresses the marginal contribution of the pilot policy, whereas the emission-reduction potential of emerging green infrastructure, such as charging stations and clean energy grids, has not yet been fully realized.

The findings above provide clear implications for differentiated low-carbon governance: greater resource allocation and policy focus should be directed towards cities with weak infrastructure to amplify their incremental gains from the pilot policy. For cities with advanced infrastructure, complementary unlocking strategies—such as retrofitting green infrastructure and upgrading industrial structures—are necessary to overcome path dependency. Therefore, Hypothesis 4 is validated.

## 5 Conclusion and discussion

### 5.1 Research conclusions

Based on the low-carbon city pilot policy and employing panel data from Chinese prefecture-level cities from 2008 to 2022, this study conducts both theoretical and empirical analyses of the relationship between the low-carbon pilot policy and urban air quality. The main findings are as follows:

First, the low-carbon city pilot policy exerts a significant positive effect on urban air quality improvement. This conclusion remains robust after a series of rigorous tests, including parallel trend tests, placebo tests, entropy balancing validation, exclusion of specific time periods and spatial samples, and excluding interference from other policies. This not only confirms the effectiveness of low-carbon pilots in environmental governance but also provides direct empirical evidence for their co-benefits in pollution reduction and carbon mitigation. Second, mechanism analysis reveals that, on one hand, the low-carbon pilot policy reduces the pollution intensity per unit of economic output at the source by enhancing green economic efficiency. On the other hand, it promotes the upgrading of industrial structure, reshaping the city’s green foundation at the macro level. The parallel operation of these two pathways reveals that the low-carbon policy does not rely solely on technological improvement or structural adjustment in isolation; rather, it couples both through institutional design to generate synergistic effects. Meanwhile, the study also finds that the level of urban infrastructure exerts a negative moderating effect on policy outcomes—that is, the more developed the infrastructure, the weaker the marginal improvement effect of the policy. This confirms the existence of a high-carbon lock-in effect: well-developed but carbon-intensive traditional infrastructure may instead become an obstacle to green transition, compressing the marginal improvement space of the policy and thereby influencing its effectiveness. Third, heterogeneity analysis indicates that the policy is more effective in non-provincial capital cities, larger cities, cities with greater fiscal investment, and cities in high-energy-consumption regions. This suggests that the implementation effectiveness of the policy is highly dependent on local governance capacity, resource endowments, economic scale, and industrial structure.

The theoretical contributions of the above findings are threefold. First, they extend the research perspective on the low-carbon pilot policy from a singular focus on carbon reduction to the synergy between pollution reduction and carbon mitigation, enriching the understanding of the comprehensive benefits of environmental regulation. Second, they identify two parallel mechanisms—green economic efficiency and industrial structure upgrading—and reveal their complementary relationship at the source and structural levels, deepening the understanding of the realization pathways of the “innovation compensation” effect within the Porter Hypothesis. Third, the discovery and validation of the negative moderating effect of infrastructure level provide new evidence from low-carbon policy practice for the theories of path dependence and lock-in effect in environmental economics, suggesting that green transformation must pay attention not only to incremental new construction but also to the challenges of existing stock retrofitting.

### 5.2 Policy recommendations

Based on the above findings, the subsequent optimization and wider promotion of the low-carbon city pilot policy should build a collaborative governance system featuring precise governmental guidance, proactive corporate transformation, and multi-stakeholder societal participation. Accordingly, the following specific policy recommendations are proposed.

For central and local governments, it is recommended to adopt differentiated implementation strategies based on the continuation of the pilot policy. For large cities and cities with stronger fiscal capacity, carbon intensity targets should be raised and the coverage of carbon markets expanded, strengthening their role in leading technological innovation. For small and medium-sized cities and cities with weaker fiscal capacity, more flexible implementation pathways should be designed to avoid a one-size-fits-all approach. Examples include reducing transition costs through central government special fiscal transfers and provincial-level green technology assistance platforms, while establishing a policy evaluation mechanism centered on air quality improvement. For cities located in high-energy-consumption regions, these cities should be prioritized as key breakthrough points for deepening low-carbon pilots. Stricter carbon emission constraints should be implemented, with higher targets set for energy efficiency improvement and carbon intensity reduction, thereby forcing them to accelerate the phase-out of outdated production capacity and promote green technological transformation. Meanwhile, high-energy-consumption cities could be encouraged to take the lead in exploring carbon trading pilots in key industries, translating emission reduction pressure into market-based incentives, thereby providing replicable experience for low-carbon transition in similar regions.

For enterprises, proactive green transformation is essential. The research confirms that green technology innovation and production process upgrading are the core pathways through which the policy generates environmental benefits. Therefore, enterprises, especially those in traditional energy-intensive industries, must recognize that environmental compliance is not merely a cost but a strategic investment crucial for long-term survival and competitiveness. Enterprises should actively utilize policy tools available in pilot areas, such as green credit, tax incentives, and technology subsidies, to increase investment in the research, development, and application of cleaner production technologies. By improving processes, substituting energy sources, and adopting circular economy models, they can reduce pollution emissions at the source. Leading enterprises should further play a demonstrative role by building green supply chains centered around themselves, extending environmental standards to upstream and downstream partners, thereby amplifying the leverage effect of individual corporate emission reductions.

For the general public, active participation in supervision and promotion is encouraged to form a soft constraint that drives policy refinement. The rise in public environmental awareness and green consumption choices can generate strong market demand signals, compelling enterprises to provide more environmentally friendly products and services. Furthermore, the public should actively pay attention to corporate environmental information disclosure and government environmental law enforcement. Through channels such as environmental complaint hotlines and government service platforms, they can provide truthful feedback and supervision regarding observed environmental issues—such as pollution emissions and ecological damage—or instances of inadequate policy implementation.

### 5.3 Research limitations and future prospects

The research period of this study is set from 2008 to 2022, aiming to comprehensively examine the medium- to long-term impacts of the low-carbon pilot policy under normalized economic conditions, while avoiding data consistency issues arising from adjustments in statistical caliber or incomplete data releases for some prefecture-level indicators after 2023. Although the data end in 2022, this study still offers valuable insights for understanding low-carbon governance in the current and future periods.

First, the period 2008–2022 precisely covers the complete cycle of the low-carbon city pilot initiative—from its initial launch and gradual expansion to its deep integration into national strategy. This period represents a critical phase during which the policy evolved from local experimentation to nationwide rollout, and its experiences and lessons constitute the cornerstone for subsequent policy optimization. Particularly after the official introduction of the “dual carbon” goals in 2020, the data from 2021 to 2022 capture the practical effects of the pilot policy in the early stages of the “dual carbon” strategy, serving as a bridge between past implementation and future adaptation, and holding significance for assessing the policy’s adaptability and effectiveness under new circumstances. Second, although the advancement of the “dual carbon” goals has continued to deepen after 2022, the realization of policy effects is characterized by long-term and lagged features. The mechanisms of policy action revealed in this study—namely, the improvement of green economic efficiency, the upgrading of industrial structure, and the lock-in effect of infrastructure—are structural factors that cannot be easily altered by short-term policy adjustments. The analytical conclusions regarding these deep-seated mechanisms therefore retain a certain guiding significance for deepening low-carbon pilots and promoting the synergistic effects of pollution reduction and carbon mitigation in the current and future contexts.

Future research can be extended in two directions based on the findings of this study. First, as prefecture-level statistical data become more refined and complete in the future, the research period can be extended to more recent years to examine the latest effects of low-carbon pilots under the deepening background of the “dual carbon” goals. Second, future studies could incorporate more micro-level data on enterprises or residents’ behavior to further explore the policy’s deeper impacts on aspects such as green technology innovation and the transition to green lifestyles, thereby presenting a more comprehensive picture of the evolutionary trajectory of low-carbon transition.

## Data Availability

The raw data supporting the conclusions of this article will be made available by the authors, without undue reservation.

## Footnotes

1 Data source: *“PM2.5 Concentration in Chinese Cities Declines for Ten Consecutive Years.”* Science and Technology Daily, April 25, 2023. (available at: http://news.cnjiwang.com/dyxw_kx/202304/3715869.html).

## References

[1] Li M Liu T Wu G Lin J Guo B. Has the development of artificial intelligence promoted urban pollutant and carbon emission reduction? Evidence from China. Front Public Health. (2026) 13:1739342. doi: 10.3389/fpubh.2025.1739342, 41607909 PMC12835399

[2] Jiang J Lin J Guo B. How does digital-green policy synergy affect substantive and strategic green technology innovation? Evidence from China. Int Rev Econ Finance. (2026) 106:104969. doi: 10.1016/j.iref.2026.104969

[3] Wang S Guo B. Impact of green finance on urban ecological and environmental resilience: evidence from China. Sustainability. (2026) 18:706. doi: 10.3390/su18020706

[4] Yan J Zhao J Yang X Su X Wang H Ran Q . Does low-Carbon City pilot policy alleviate urban haze pollution? Empirical evidence from a quasi-natural experiment in China. IJERPH. (2021) 18:11287. doi: 10.3390/ijerph182111287, 34769802 PMC8583181

[5] Tang P Yang S Shen J Fu S. Does China’s low-carbon pilot programme really take off? Evidence from land transfer of energy-intensive industry. Energy Policy. (2018) 114:482–91. doi: 10.1016/j.enpol.2017.12.032

[6] Wen S Jia Z Chen X. Can low-carbon city pilot policies significantly improve carbon emission efficiency? Empirical evidence from China. J Clean Prod. (2022) 346:131131. doi: 10.1016/j.jclepro.2022.131131

[7] Chen L Wang K. The spatial spillover effect of low-carbon city pilot scheme on green efficiency in China’s cities: evidence from a quasi-natural experiment. Energy Econ. (2022) 110:106018. doi: 10.1016/j.eneco.2022.106018

[8] Liu B Gan L Huang K Hu S. The impact of low-carbon city pilot policy on corporate green innovation: evidence from China. Finance Res Lett. (2023) 58:104055. doi: 10.1016/j.frl.2023.104055

[9] Qiu S Wang Z Liu S. The policy outcomes of low-carbon city construction on urban green development: evidence from a quasi-natural experiment conducted in China. Sustain Cities Soc. (2021) 66:102699. doi: 10.1016/j.scs.2020.102699

[10] Rennings K. Redefining innovation — eco-innovation research and the contribution from ecological economics. Ecol Econ. (2000) 32:319–32. doi: 10.1016/S0921-8009(99)00112-3

[11] Wang F Ge X. Does low-carbon transition shock employment? Empirical evidence from the low-carbon city pilot initiative. China Industrial Economics. (2022) 5:81–99. doi: 10.19581/j.cnki.ciejournal.2022.05.004

[12] Kemp R Pontoglio S. The innovation effects of environmental policy instruments — a typical case of the blind men and the elephant? Ecol Econ. (2011) 72:28–36. doi: 10.1016/j.ecolecon.2011.09.014

[13] Testa F Iraldo F Frey M. The effect of environmental regulation on firms’ competitive performance: the case of the building & construction sector in some EU regions. J Environ Manag. (2011) 92:2136–44. doi: 10.1016/j.jenvman.2011.03.039, 21524840

[14] Essamlali I Nhaila H Khaili ME. Impact of urban block shape on traffic and air quality: a SUMO-based comparative study of rectangular, radial, and triangular forms. Trans. Res. Interdiscip. Perspec. (2025) 31:101413. doi: 10.1016/j.trip.2025.101413

[15] Augusto B Coelho S Rafael S Coelho MC Ferreira J. How does urban morphology impact cities air quality? A modelling study. Sci Total Environ. (2025) 973:179138. doi: 10.1016/j.scitotenv.2025.179138, 40112549

[16] Zhang P Zhang J Liu Z Liu Y Chen Z. Relationship between land surface temperature and air quality in urban and suburban areas: dynamic changes and interaction effects. Sustain Cities Soc. (2025) 118:106043. doi: 10.1016/j.scs.2024.106043

[17] Ambrey CL Fleming CM Chan AY-C. Estimating the cost of air pollution in south East Queensland: an application of the life satisfaction non-market valuation approach. Ecol Econ. (2014) 97:172–81. doi: 10.1016/j.ecolecon.2013.11.007

[18] Schikowski T Vossoughi M Vierkötter A Schulte T Teichert T Sugiri D . Association of air pollution with cognitive functions and its modification by APOE gene variants in elderly women. Environ Res. (2015) 142:10–6. doi: 10.1016/j.envres.2015.06.009, 26092807

[19] Furie GL Balbus J. Global environmental health and sustainable development: the role at Rio+20. Ciênc saúde coletiva. (2012) 17:1427–32. doi: 10.1590/S1413-81232012000600007, 22699634

[20] Greenstone M Hanna R. Environmental regulations, air and water pollution, and infant mortality in India. Am Econ Rev. (2014) 104:3038–72. doi: 10.1257/aer.104.10.3038

[21] Zhao C Wang B. How does new-type urbanization affect air pollution? Empirical evidence based on spatial spillover effect and spatial Durbin model. Environ Int. (2022) 165:107304. doi: 10.1016/j.envint.2022.107304, 35640449

[22] Acemoglu D Aghion P Hemous D. The environment and directed technical change in a north-south model. Oxf Rev Econ Policy. (2014) 30:513–30. doi: 10.1093/oxrep/gru031 PMC4692283 26719595

[23] Tokimatsu K Konishi S Ishihara K Tezuka T Yasuoka R Nishio M. Role of innovative technologies under the global zero emissions scenarios. Appl Energy. (2016) 162:1483–93. doi: 10.1016/j.apenergy.2015.02.051

[24] Song H Sun Y Chen D. Assessment of the government’s air pollution control effect: an empirical study from the construction of “low-carbon cities” in China. J Manag World. (In Chinese). (2019) 35:95–108+195.

[25] Gehrsitz M. The effect of low emission zones on air pollution and infant health. J Environ Econ Manag. (2017) 83:121–44. doi: 10.1016/j.jeem.2017.02.003

[26] Lu Z Xu M Shi L Lei T. The criticality of environmental sustainability in agriculture: the decarbonization role of green finance in China. J Environ Manag. (2025) 394:127470. doi: 10.1016/j.jenvman.2025.127470 41038104

[27] Zhang J Zheng T. Can dual pilot policy of innovative city and low carbon city promote green lifestyle transformation of residents? J Clean Prod. (2023) 405:136711. doi: 10.1016/j.jclepro.2023.136711

[28] Zang C Sun P. Does the construction of low-carbon cities promote local green development? Empirical evidence from a quasi-natural experiment. Finance Trade Res. (In Chinese). (2021) 10:27–40.

[29] Huo W Qi J Yang T Liu J Liu M Zhou Z. Effects of China’s pilot low-carbon city policy on carbon emission reduction: a quasi-natural experiment based on satellite data. Technol Forecast Soc Change. (2022) 175:121422. doi: 10.1016/j.techfore.2021.121422

[30] Lu Z Gou D Yang L Wu Z Feng H. The impact of environmental tax reform on industrial green development: evidence from China. Front Environ Sci. (2025) 13:1593549. doi: 10.3389/fenvs.2025.1593549

[31] Qi S Zhou C Li K Tang S. The impact of a carbon trading pilot policy on the low-carbon international competitiveness of industry in China: an empirical analysis based on a DDD model. J Clean Prod. (2021) 281:125361. doi: 10.1016/j.jclepro.2020.125361

[32] Porter ME Linde CVD. Toward a new conception of the environment-competitiveness relationship. J Econ Perspect. (1995) 9:97–118. doi: 10.1257/jep.9.4.97

[33] Tariq A Badir YF Tariq W Bhutta US. Drivers and consequences of green product and process innovation: a systematic review, conceptual framework, and future outlook. Technol Soc. (2017) 51:8–23. doi: 10.1016/j.techsoc.2017.06.002

[34] Fu Y He C Luo L. Does the low-carbon city policy make a difference? Empirical evidence of the pilot scheme in China with DEA and PSM-DID. Ecol Indic. (2021) 122:107238. doi: 10.1016/j.ecolind.2020.107238

[35] Hu J Zhang H Irfan M. How does digital infrastructure construction affect low-carbon development? A multidimensional interpretation of evidence from China. J Clean Prod. (2023) 396:136467. doi: 10.1016/j.jclepro.2023.136467

[36] Wang M Guo B. Have industrial robots reduced carbon emissions? Empirical evidence from China. Technol Soc. (2026) 86:103244. doi: 10.1016/j.techsoc.2026.103244

[37] Lv T Hu H Zhang X Xie H Wang L Fu S. Spatial spillover effects of urbanization on carbon emissions in the Yangtze River Delta urban agglomeration, China. Environ Sci Pollut Res. (2022) 29:33920–34. doi: 10.1007/s11356-021-17872-x, 35031992

[38] Chastko K Adams M. Improving long-term air pollution estimates with incomplete data: a method-fusion approach. MethodsX. (2019) 6:1489–95. doi: 10.1016/j.mex.2019.06.005, 31297334 PMC6597923

[39] Shao S Li X Cao J. China’s economic policy choices for haze pollution control: from the perspective of spatial spillover effects. Econ Res J. (In Chinese). (2016) 51:73–88.

[40] Fang X Tian S Wang X. Fiscal decentralization, industrial agglomeration, and green economic efficiency: an empirical analysis based on panel data from 270 prefecture-level and above cities in China. Inquiry into Econ Issues. (In Chinese). (2019) 18:164–72.

[41] Fang L Tang H Li J. The impact of National Independent Innovation Demonstration Zone Construction on urban air pollution: evidence from a quasi-natural experiment. J Xi’an Jiaotong Univ. (In Chinese). (2022) 45:49–62.

[42] Cantoni D Chen Y Yang DY Yuchtman N Zhang YJ. Curriculum and ideology. J Polit Econ. (2017) 125:338–92. doi: 10.1086/690951

[43] Yu X Lin J. The impact of low-carbon pilot policy on atmospheric environment and its spatial effects. Chinese J Environ Management. (In Chinese). (2023) 15:56–70. doi: 10.16868/j.cnki.1674-6252.2023.06.056

[44] Baron RM Kenny DA. The moderator–mediator variable distinction in social psychological research: conceptual, strategic, and statistical considerations. J Pers Soc Psychol. (1986) 51:1173–82. doi: 10.1037/0022-3514.51.6.1173 3806354

